# A Patient Loses 18 kg and Type 2 Diabetes Mellitus: The Challenge of Complete Remission

**DOI:** 10.7759/cureus.4817

**Published:** 2019-06-03

**Authors:** Samih A Odhaib, Abbas A Mansour

**Affiliations:** 1 Diabetes and Adult Endocrinology, Faiha Specialized Diabetes, Endocrine and Metabolism Center, College of Medicine, University of Basrah, Basrah, IRQ; 2 Diabetes and Endocrinology, College of Medicine, University of Basrah, Basrah, IRQ

**Keywords:** diabetes, diabetes in remission, complete remission, weight loss

## Abstract

Type 2 diabetes mellitus (T2DM) in adulthood is strongly related to weight gain and excessive accumulation of pancreatic and hepatic fat. We present a case of a 48-year-old man with diabetes who presented two years prior with poorly controlled T2DM diagnosed more than three years before presentation. He had severe hypertension and marked dyslipidemia. He underwent a complete remission of his diabetes after nonintentionally losing 18 kg (his original weight: 58 kg). The patient had excellent euglycemic measures on no treatment at all for the last two years with healthy blood pressure and lipid profile and a reduced 10-year risk of heart disease and stroke from 11% to 3.3%. This case demonstrates the effect of weight loss on glycemic control and consequent remission in T2DM patients.

## Introduction

The development of type 2 diabetes mellitus (T2DM) in adulthood is strongly related to weight gain and excessive accumulation of pancreatic and hepatic fat [1]. Remission of T2DM in obese diabetic patients is very uncommon without bariatric surgery [2]. An acute negative energy balance can reverse the twin defects of beta-cell failure and insulin resistance with a diet restriction to around 700 kcal [1, 3-4]; that results in decreased pancreatic and liver triacylglycerol stores [3]. The change in peripheral insulin sensitivity played no part in the early return of normoglycemia [3]. Lean et al. conducted one of the more valuable studies in this field and demonstrated the effect of weight loss on the long-term course of T2DM, reporting that T2DM remission occurred in approximately half the sample patients [1]. Here, we present a case of a lean patient with T2DM with other comorbidities who achieved complete remission of T2DM by weight loss only.

## Case presentation

A 46-year-old man presented to Faiha Specialized Diabetes, Endocrine, and Metabolism Center (FDEMC) in late December 2016 for poorly controlled T2DM (diagnosed in June 2014) and hypertension (diagnosed in August 2015). He had been unable to pay for his medications or investigations, which prevented him from achieving his treatment goals. He received no treatments for two years prior to presentation, and he unsuccessfully attempted to manage his diabetes via diet. The patient is married and smokes tobacco. He has a family history of T2DM and ischemic heart disease. 

The results of his cardiorespiratory, abdominal, head and neck, and ophthalmological examinations were within reference ranges. He had a mild bilateral weakness and paresthesia involving the distal parts of the hands and feet not following any pattern. He weighed 58 kg; other examination findings are listed in Table 1.

**Table 1:** Clinical examination findings on initial presentation. ASCVD, atherosclerotic cardiovascular disease; ACC, American College of Cardiology; AHA, American Heart Association.

Characteristic/Analyte	Measurement
Weight (kg)	58
Height (cm)	166
Body mass index	21.05 kg/m^2^
Blood pressure	140/90 mmHg
Glycosylated hemoglobin	11.2%
Fasting plasma glucose	175 mg/dL (9.72 mmol/L)
Triglycerides	393 mg/dL (4.44 mmol/L)
Total cholesterol	255 mg/dL (6.6 mmol/L)
High-density lipoprotein-cholesterol	46 mg/dL (1.19 mmol/L)
Low-density lipoprotein-cholesterol	155 mg/dL (4.01 mmol/L)
Liver and renal function tests	Within reference ranges
Thyroid function tests	Within reference ranges
Complete blood count	Within reference ranges
Urine and electrolytes	Within reference ranges
10-year risk of heart disease and stroke (ASCVD/ACC/AHA heart risk calculation)	11%

We advised the patient to begin insulin therapy and a carefully controlled dietary plan, but he refused the insulin therapy. Accordingly, we recommended oral therapy by metformin (1000 mg/day) with vildagliptin (50 mg/day), atorvastatin (20 mg/day in the evening), and captopril (50 mg/day).

At the three-month follow-up examination, the patient’s glycosylated hemoglobin (HbA1c) was elevated (10.8%); his blood pressure was 160/100 mmHg, with no other changes from the initial examination. His fasting plasma glucose ranged from 187 to 295 mg/dL (10.39-16.39 mmol/L). We again recommended insulin therapy, but he declined. We provide him with a free one-month supply of vildagliptin/metformin combined tablet (50/1000 mg) from the Center, and we urged him to continue his other therapies at the same doses along with improved dietary control. The patient was lost to follow-up for the next 20 months.

After 20 months, the patient presented again and reported he quit all medications one week after his second visit to FDEMC. He reported that he relied solely on diet control due to financial restrictions. His return to the clinic was prompted by his concerns regarding all-day musculoskeletal pain, fatigue, palpitation, and erectile dysfunction lasting five months. On examination, he was ill-looking, cachectic, with a darkened complexion and marked weight loss (with a scaphoid abdomen). His weight was 41 kg, and his BMI had dropped to 14.9 kg/m2. His blood pressure was 116/68 mmHg (supine), 90/44 mmHg (standing), with a postural drop of 26/24 mmHg, and his resting pulse rate was 116 beats per minute. There was a diffuse goiter, with bruit, no lymphadenopathy, and no compressive symptoms. Table 2 presents the results of his examination.

**Table 2:** Clinical examination results after 20-month loss to follow-up.

Examination	Result
Electrocardiogram and chest radiography	Normal
Thyroid stimulating hormone	<0.01 mIU/L
Total thyroxine	24.86 μg/dL (320 nmol/L)
Free thyroxine	4.3 ng/dL (55.35 pmol/L)
Fasting plasma glucose	80 mg/dL (4.4 mmol/L)
Glycosylated hemoglobin	5.6%
Lipid profile, renal function, liver function panel, and urine and electrolytes	Within reference ranges
Complete blood count, erythrocytes sedimentation rate, C-reactive protein	Within reference ranges
Anti-tissue transglutaminase antibody IgA	0.5 U/mL
Total testosterone, sex hormone binding globulin, free testosterone	Within reference ranges

The results of our examinations were somewhat troubling and bore repeating for confirmation. Table 3 presents the results of the patient’s laboratory studies.

**Table 3:** Clinical laboratory examination.

Analyte	Result
Fasting plasma glucose	76 mg/dL (4.2 mmol/L)
Glycosylated hemoglobin	5.7%
Triglycerides	91 mg/dL (1.03 mmol/L)
Total cholesterol	131 mg/dL (3.39 mmol/L)
High-density lipoprotein-cholesterol	46 mg/dL (1.19 mmol/L)
Low-density lipoprotein-cholesterol	56 mg/dL (1.45 mmol/L)
Very-low-density lipoprotein-cholesterol	20 mg/dL (0.52 mmol/L)
Non-high-density lipoprotein-cholesterol	60 mg/dL (1.55 mmol/L)
Thyroid stimulating hormone	<0.001 mIU/L
Free thyroxine	>7.7 ng/dL (> 100 pmol/L)
Total thyroxine	37 μg/dL (476.26 nmol/L)
Total triiodothyronine	>650 ng/dL (10 nmol/L)
Anti-thyroid peroxidase antibody	585 IU/mL
Thyroid receptor antibody	41.14 IU/mL (NV<1.75 mIU/mL)
Thyroglobulin	10.25 ng/mL
Parathyroid hormone	13.53 pg/mL (1.44 pmol/L)
25-hydroxycholecalciferol	22 ng/mL (55 nmol/L)
Anti-tissue transglutaminase antibody IgA	0.5 U/mL
Short synacthen stimulation test	Normal
C-peptide level	1.73 ng/mL (0.57 nmol/L)
Insulin level	5.72 mIU/L (39.72 pmol/L)
Anti-glutamic acid decarboxylase antibody	Negative
Total testosterone, sex hormone binding globulin, free testosterone	Within reference ranges

An ultrasound of the patient’s largest right thyroid lobe revealed a nodule (19.4 mm x 30.0 mm) with a surrounding hypoechoic halo and tiny internal foci of calcification.

The patient lost approximately 18 kg over 20 months, which constituted more than 30% of his original weight, during which he received no treatment for his diabetes, hypertension, and dyslipidemia. The patient developed Grave’s disease. We started treatment with carbimazole (45 mg/day) with a multivitamin formula and provided smoking cessation coaching. The patient was discharged with instructions to return in six weeks for a follow-up examination.

His 10-year risk of heart disease or stroke had dropped to 3.3% and would be further reduced to 1.4% if he quit smoking. The patient’s diabetes is in complete remission according to the American Diabetes Association (ADA) Consensus Statement, which defines complete remission as euglycemia measures lasting at least one year while the patient is on no active pharmacologic therapy or ongoing procedures [5].

In early 2019, the patient successfully quit smoking and had gained about 2 kg in body weight. He also presented with a better physique and detectable thyroid stimulating hormone of 0.01 mIU/L. However, he had a free thyroxine reading of >7.77 ng/dL (>100 pmol/L). His fasting glucose was 91 mg/dL (5.06 mmol/L). A second ultrasound of the thyroid showed no changes from the previous ultrasound. In the next two months, the patient continued the same dose of carbimazole (45 mg/day) until achieving the euthyroid status.

In March 2019, fine needle aspiration of the largest nodule revealed a Bethesda II hyperplastic nodule. The patient had developed dysphagia and compressive symptoms, and was scheduled for thyroidectomy. He underwent a near-total thyroidectomy with an excellent noneventful convalescence period. We initiated levothyroxine (100 µg/ day) according to his postoperative investigations, with euglycemic measures and the same preoperative body weight.

## Discussion

According to the Lean et al., a weight loss of at least 10-15 kg leads to euglycemia in people with short-term T2DM for at least 12 months. However, T2DM lasting up to six years is not necessarily a permanent, lifelong condition [1].

Weight loss leads to a rapid and marked fall in blood pressure, with a risk of postural hypotension on pressure-reducing medications. The low-energy formula produces a blood pressure fall that is greater than that achieved from the reduction of salt intake alone [1]. Regarding lipids, we expect triglyceride and cholesterol concentrations to be lower than expected from guideline-driven statin prescriptions [1].

High rates of remission of T2DM are associated with several factors including advanced age, being African American, having higher socioeconomic status, renal impairment, and an absence of dyslipidemia [2]. Baseline BMI is an independent factor and does not affect the T2DM remission rate in obese patients [6]. Although the thyrotoxicosis alone is diabetogenic [7], no relationship exists between the duration of diabetes or any diabetes-related variables and the presence of thyroid dysfunction [8]. However, some studies revealed 10% lower HbA1c levels in patients with hyperthyroid diabetes in comparison to patients with euthyroid diabetes [8]. In untreated Graves’ disease, increased proinsulin levels in response to a meal with a reduced C-peptide to proinsulin ratio suggested an underlying defect in proinsulin processing. The excess thyroid hormones may facilitate the glucose absorption from the gut, and stimulate gluconeogenesis [9-10]. A simultaneous disturbance of pancreatic and thyroidal metabolism deteriorates metabolic control and residual beta-cell function [11].

The remission of T2DM in obese patients differs from the remission in lean diabetic patients who develop massive weight loss or cachexia because cachexia may have a different metabolic effect on glycemic control. Diabetes remission here may be the effect of severe weight loss and loss of both muscle and fat mass due to the inanition effect. In such a condition, glucose becomes the sole oxidative fuel in the setting of total body fat and muscle depletion, and the resulting high rates of glucose utilization exceed the capacity to produce glucose by glycogenolysis and gluconeogenesis because of a limited supply of gluconeogenic precursors (e.g., amino acids). What can cause hypoglycemia in nondiabetics may induce remission in diabetic patients [12-13].

We used the 2013 version of the Atherosclerotic Cardiovascular Disease (ASCVD) risk score from the American College of Cardiology instead of the broader 2017 version in assessing our patients’ 10-year cardiovascular risk after remission because the 2017 version cannot estimate the ASCVD risk for patients with the low-density lipoprotein-C of <70 mg/dL (1.81 mmol/L) [14].

The ADA consensus statement on the treatment of comorbid conditions in patients in complete or partial remission is the same for patients with current T2DM, even for the possible microvascular complications screening [5].

## Conclusions

This case highlights the fact that complete remission of T2DM is possible by a weight loss of >15 kg. More data and follow-up are needed for ensuring long-term or permanent remission of DM with continued monitoring for at least five years.
